# Simulation training for emergency skills: effects on ICU fellows’ performance and supervision levels

**DOI:** 10.1186/s12909-020-02419-4

**Published:** 2020-12-09

**Authors:** Bjoern Zante, Joerg C. Schefold

**Affiliations:** Department of Intensive Care Medicine, Inselspital, Bern University Hospital, University of Bern, Freiburgstrasse 10, 3010 Bern, Switzerland

**Keywords:** EPA, Chest tube, Pericardiocentesis, Cricoconiotomy, Education, Learning, Critical care, Cost effective, Assessment, Self perception

## Abstract

**Background:**

The application of manual emergency skills is essential in intensive care medicine. Simulation training on cadavers may be beneficial. The aim of this study was to analyze a skill-training aiming to enhance ICU-fellows´ performance.

**Methods:**

A skill-training was prepared for chest tube insertion, pericardiocentesis, and cricothyroidotomy. Supervision levels (SL) for entrustable professional activities (EPA) were applied to evaluate skill performance. Pre- and post-training, SL and fellows´ self- versus consultants´ external assessment was compared. Time on skill training was compared to conventional training in the ICU-setting.

**Results:**

Comparison of pre/post external assessment showed reduced required SL for chest tube insertion, pericardiocentesis, and cricothyroidotomy. Self- and external assessed SL did not significantly correlate for pre-training/post-training pericardiocentesis and post-training cricothyroidotomy. Correlations were observed for self- and external assessment SL for chest tube insertion and pre-assessment for cricothyroidotomy. Compared to conventional training in the ICU-setting, chest tube insertion training may further be time-saving.

**Conclusions:**

Emergency skill training separated from a daily clinical ICU-setting appeared feasible and useful to enhance skill performance in ICU fellows and may reduce respective SL. We observed that in dedicated skill-training sessions, required time resources would be somewhat reduced compared to conventional training methods.

## Background

Intensive care physicians should be familiar with emergency skill procedures such as chest tube insertion, pericardiocentesis and cricothyroidotomy. In environments with time constraints such as intensive care units, education may compete with clinical work and administrative responsibilities [1]. Therefore, educational concepts should be applied with specific and clear learning objectives [2]. Further, teaching and learning of technical skills in the ICU environment seems determined by caseload and, particularly for emergency skills, even more by suitable teaching cases in respective (emergency) situations. The transfer of training from the clinical setting to “safe environments” such as training on human cadavers in a wet lab may have beneficial effects on teaching/ learning experiences [3–6].

For previous familiarization with the theoretical content, the flipped classroom concept seems beneficial to focus on skill performance in subsequent trainings [7, 8]. In brief, in the flipped classroom concept, learners are familiarized to the educational content and in subsequent training they can apply new knowledge. In this concept, the andragogy theory of involves principles of adult learning [9]. Furthermore, the constructivism theory describes the learner as the architect of his own knowledge [10]. The flipped classroom concept ensures to adapt the volume of learning time, aligned by the learners’ needs and previous information.

Importantly, based on Simpson’s [11] and Harrow’s [12] taxonomy of psychomotor domains a technical skill are developed continuously over 5 stages: 1) guided response, imitation or try and error, 2) skills become habitual, 3) complex overt response, quick and accurate performance, 4) adaption with the ability to modify skill in difficult situations, 5) skill has mastered and new movements addressed unique situations and specific problems [13]. Especially for step one (with regard to emergency skills) “try and error” should be avoided in direct patient care and should thus be performed in simulated training.

Typically, skill training covers two phases with five steps: first, a cognitive phase: step 1) learning should be performed via reading and visualization (e.g. video) and step 2) viewing demonstration of skills [13, 14]. Phase two, the psychomotor phase, includes step 3) formative assessment on simulator training to deliberate practice, 4) summative assessment on simulator training and finally step 5) performance on patients [13, 14].

Secure performance on actual patients without need for direct supervision should thus be a key objective of skill training. Hence, skill training should have a positive effect on fellows’ performance and may increase trust to reduce subsequent supervision levels in daily practice. The aim of the current investigation was to analyze a new emergency skill training program to enhance the performance of ICU fellows. In particular, we were interested whether such a program would reduce supervision levels, based on external assessment.

## Methods

### Participants

Participants of the new skill training program were ICU-fellows in a tertiary care academic hospital (Inselspital, University of Bern, Switzerland). Every year about 6500 patients are treated in our multidisciplinary Department of Intensive Care Medicine. The department compromises about 57 beds and treats all types of (multi) organ failures, including extracorporeal membrane oxygenation (ECMO). ICU-fellows in this investigation are typically subjected to > 12 months of training in intensive care medicine. Within this training program, full day skill training was performed twice per year since 2017. Over this period, 31 ICU-fellows participated in the skill-training program and were invited to participate in this voluntary assessment that also aimed to assess the quality of the established training program. This assessment adheres to the declaration of Helsinki.

### Educational concepts: design and setting

Learning objectives in this skill-training program consisted in skill technics considered highly relevant for emergency situations in the ICU setting: ICU-fellows were trained in chest tube insertion, pericardiocentesis, and cricothyroidotomy. Training on human cadavers was performed in the laboratory of the Institute of Anatomy, Faculty of Medicine, Bern, Switzerland. Cadaver training was designed to improve confidence in the skill performance [4, 5, 15–19]. Particular for emergency skill training, and when compared to artificial skill-simulators, cadavers may provide improved learning experiences [4–6]. Pre-training, all fellows received literature and video documents along with the departmental standard operating procedures (SOP), including previously published articles/ video training [20–23]. Fellows prepared the obtained learning material individually prior to skill training. A maximum of six fellows with two experienced attending consultants (teachers) applied in an effort to maximize learning experiences and provide best individual support [24]. Formative assessment during training was performed to enhance fellows’ skill performance. A summative assessment was performed with the consultants’ post-training assessment of required supervision levels to determine future required supervision levels for daily ICU work.

### Assessment of fellows’ self- and external perception

The five supervision levels are based on Ten Cates´ levels for entrustable professional activities [25]. In brief, supervision levels are defined as followed: 1) Observation but not execution, even with direct supervision, 2) execution with direct, proactive supervision, 3) execution with reactive supervision, i.e., on request and quickly available, 4) supervision at a distance and/or post hoc and 5) supervision provided by the trainee to colleagues [25]. Participating fellows assess their expected supervision level for each skill before and after skill trainings. Based on observations in daily clinical setting prior the training, teaching consultants assessed required supervision levels of each skill for each individual fellow. After skill training, consultants re-assessed supervision levels for each skill of each individual fellow.

### Statistical analysis

Statistical analysis was performed using MedCalc 17.4 (MedCalc Software, Ostend, Belgium). Wilcoxon rank sum test was used to compare pre- and post-training (perceived) supervision levels. Spearman rank correlations were used to compare fellows´ and consultants´ perceptions of supervision level. A descriptive analysis was aimed for. A two-tailed *p*-value < 0.5 was considered significant.

## Results

Data from self-assessment surveys (expected supervision levels of respective skill) was available in 94% (29/31) of cases.

### Assessment of chest tube insertion

**Fig. 1:**
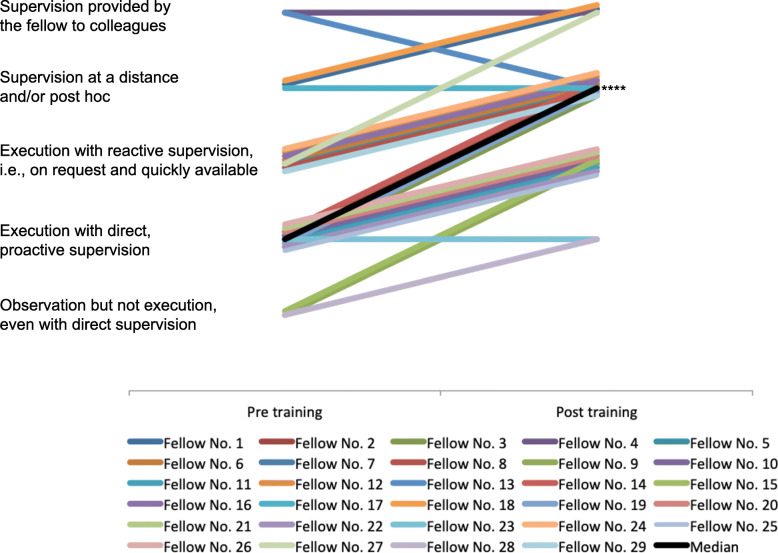
Chest drain insertion – external perception. ****) *p* < 0.0001 for median changes in supervision levels

Results of pre−/ post-training self- and external assessment are given (Table 1A). Self-assessment of 21 fellows (21/29, 72%) rated a positive difference between pre- and post-training supervision (“Execution with reactive supervision, i.e., on request and quickly available” to “supervision at a distance and/or post hoc”, *p* < 0.0001). External assessment rated 25 fellows (25/29, 86.21%) with an improvement on their required supervision levels (median change from “Execution with direct, proactive supervision” to “Supervision at a distance and/or post hoc”, *p* < 0.0001, Fig. 1). Self- and external assessed pre-training/post-training supervision levels were observed to correlate (r = 0.65, 95%-CI 0.37–0.82, *p* = 0.0002; r = 0.46, 95%-CI 0.1–0.71, *p* = 0.01, respectively).

**Table 1:** Results of self and external perception of supervision levels

	Self perception		External perception	
	Pre training	Post training	Pre training	Post training
**A - Chest tube insertion**				
Lowest value SL	2	3	1	2
Highest value SL	5	5	5	5
Median SL	3	4	2	4
95% CI for median SL	3.0–4.0	4.0–4.23	2.0–3.0	3.0–4.0
IQR	2.0–4.0	3.0–5.0	2.0–3.25	3.0–4.0
Positive difference	21/29 (72%)		24/29 (83%)	
No difference	8/29 (28%)		5/29 (17%)	
Negative difference	0/29 (0%)		1/29 (3)%)	
*P*-value	0.0001		< 0.0001	
**B - Pericardiocentesis**				
Lowest value SL	1	2	1	2
Highest value SL	3	4	2	4
Median SL	1	2	1	3
95% CI for median SL	1.0–1.0	2.0–2.23	1.0–1.0	2.0–3.0
IQR	1.0–1.25	2.0–3.0	1.0–1.0	2.0–3.0
Positive difference	26/29 (90%)		29/29 (100%)	
No difference	3/29 (10%)		0/29 (0%)	
Negative difference	0/29 (0%)		0/29 (0%)	
P-value	< 0.0001		< 0.0001	
**C -** Cricothyroidotomy				
Lowest value SL	1	2	1	2
Highest value SL	5	5	2	4
Median SL	2	3	1	3
95% CI for median SL	1.0–2.0	2.0–4.0	1.0–1.0	2.0–3.0
IQR	1.0–2.0	2.0–4.0	1.0–1.25	2.0–3.0
Positive difference	22/29 (76%)		29/29 (100%)	
No difference	7/29 (24%)		0/29 (0%)	
Negative difference	0/29 (0%)		0/29 (0%)	
P-value	< 0.0001		< 0.0001	

*SL* supervision level, *CI* confidence interval, *IQR* (25th–75th) Interquartile range; Range of supervision level 1–5: 1) Observation but not execution, even with direct supervision, 2) execution with direct, proactive supervision, 3) execution with reactive supervision, i.e., on request and quickly available, 4) supervision at a distance and/or post hoc and 5) supervision provided by the trainee to colleagues

### Assessment of pericardiocentesis

**Fig. 2:**
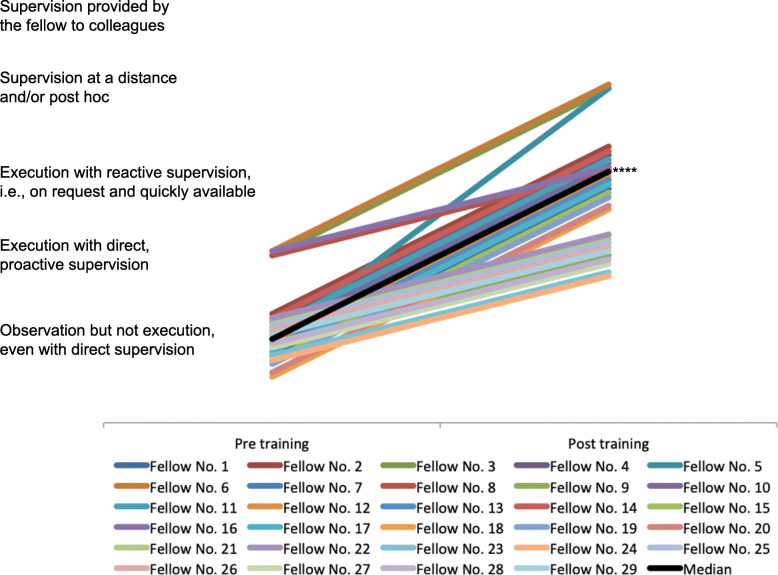
Pericardiocentesis – external perception. ****) *p* < 0.0001 for median changes in supervision levels

Results of pre−/ post-training self- and external perception are given in Table 1B. Twenty-six fellows (26/29, 90%) rated a positive effect on their expected supervision level (“Observation but not execution, even with direct supervision” to “execution with direct, proactive supervision”, *p* < 0.0001). External assessment rated 29 fellows (100%) with a positive change in their required supervision level (median change from “Observation, but not execution, even with direct supervision” to “Execution with reactive supervision, i.e., on request and quickly available”, p < 0.0001, Fig. 2). Self- and external assessed pre-training/post-training supervision level did not significantly correlate (n.s.).

### Assessment of cricothyroidotomy

**Fig. 3:**
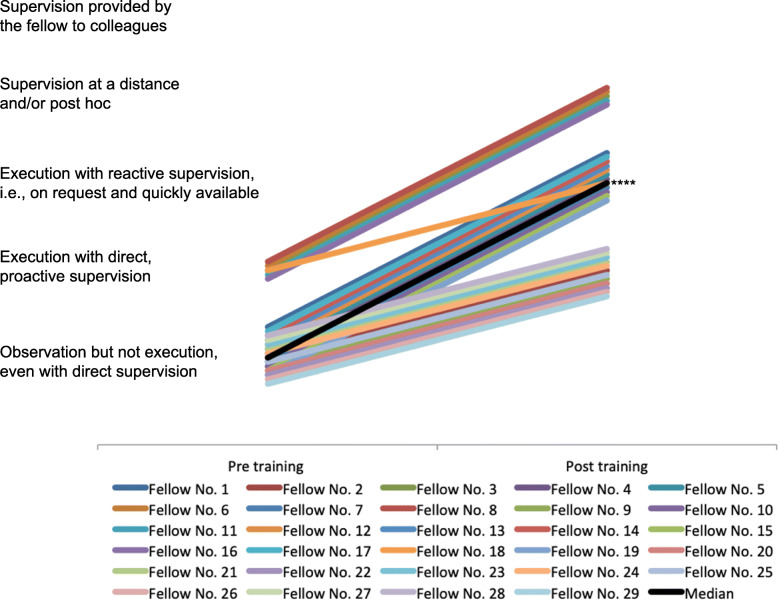
Cricothyroidotomy – external percpetion. ****) *p* < 0.0001 for median changes in supervision levels

Results of pre- /post-training perception of self and external are given in Table 1C. Twenty-two fellows (22/29, 76%) rated a positive effect on their expected supervision level (“Observation but not execution, even with direct supervision” to “execution with reactive supervision, i.e., on request and quickly available”, *p* < 0.0001). External assessment rated 29 fellows (100%) with a positive change in their supervision level (median change from “Observation but not execution, even with direct supervision” to “Execution with reactive supervision, i.e., on request and quickly available”, p < 0.0001, Fig. 3). Self-assessment of the fellows expected supervision pre-training supervision level correlated significantly with the external assessment of the required supervision level (r = 0.41, *p* = 0.03, 95%-CI 0.06–0.68). Post-training self- and external assessment of supervision level was not observed to significantly correlate (n.s.).

### Expenditure of time for emergency skill training versus clinical teaching

The part of chest tube insertion in the skill training required about two hours, in which every fellow had the opportunity to perform 6–8 chest tube insertions. In total, about 12 h (6 fellows x about 2 h) of working time applied for chest tube insertion training. For attending consultants, about 4 h of working time was required for the chest tube insertion training (about 2 × 2 h). In comparison, the time for teaching in a daily ICU-setting for each chest tube insertion required would typically be an estimated 30 min (minimum). Hence, six chest tube insertions for each fellow (about 30 min × 6 = 180 min), for six fellows (about 180 min × 6 fellows) require 1080 min (18 h) of time on supervision. During respective supervision activities, the attending consultant would be absorbed and unable to perform other activities. Hence, potential time saving for attending consultants might be about 12 h.

## Discussion

The application of emergency skills is essential in intensive care medicine. In this assessment of a newly developed training program, trust and performance was measured according to Ten Cates’ EPA levels of supervision [25]. We deliberately combined formative and summative (external) assessments to increase fellows’ performance during the training [26] and required supervision levels, respectively [13, 14]. Our observations suggest that a full day skill training may increase the ability of ICU-fellows to perform technical emergency skills such chest tube insertion, pericardiocentesis and cricothyroidotomy based on the (somewhat subjective) determination of supervision levels. Further, the opportunity to repeat respective skills during training [11, 12] and to receive feedback [27] may additionally support respective skills. The repeated practical exercise may seem key to obtain confidence and proficiency [13].

Self-assessments of ICU fellows showed a positive progression in all skills. Accurate self-assessment or self-perception of confidence and/or competence is key to ensure a sufficient learning process [28]. However, self-assessment has several limitations and may not reflect actual, i.e. objective performance [29–31]. Self-deception, i.e. lack of insight into one’s incompetence and reduced impression management [32] may limit valid self-assessment. Despite these limitations, self-assessed progression may spark the fellows’ interest and likely increase their motivation. This motivation may be key for education in ICU-residents [2] and may result in increased performance [33, 34]. Interestingly, improved learners’ (fellows) skill competence helped to acknowledge skill limitations and may lead to lower ratings in respective self-assessments [29]. Evaluating self-perceived skill competence may provide learner motivation in improving respective skills, and this may be crucial in self-efficacy regarding learning [35].

Despite the known limitations of self-assessment, for chest tube insertion self- and external assessment, expected/ required supervision levels correlated significantly. However, differences were observed in pre- and post-assessment for self and external assessment as noted for pericardiocentesis. Interestingly, post-training external assessment showed better improvement in performance (regarding supervision levels) when compared to ICU fellows’ self-assessment, which may be due to several reasons. Feedback and formative assessment during the skill training may increase skill performance without better self-perception, reduced self-deception and increased impression management [31, 36, 37]. Inadequate feedback [38] or fellows’ information neglect and memory bias may influence self-assessment [31]. Experienced fellows impression management may decrease, and self-assessment may be comparable to external assessment [31], which may open new avenues for future investigations.

Congruent pre-training self- and external assessment, as observed for cricothyroidotomy, changed into biased post self and external assessment for supervision levels. This seems in line with previous findings [29, 30] and may be explained by a realistic impression management prior to skill training due to prior experience/ training. The difference in post-training assessment may be caused by increased self-deception after the skill trainings.

### Limitations

This analysis has several additional important limitations that deserve discussion, including a limited sample size. Further, a monocentric observational investigation applied, with all inherent limitations driven by design of the investigation. Hence, an interpretation of the data should only be done with caution and further investigations with larger sample-sizes should be performed to enhance external validity. In addition, external assessment of fellows’ performance was not carried out using checklists and inter-individual assessment bias may apply. However, external assessment focused on adherence to correct technical performance as outlined in the pre-training preparation literature (including videos). However, checklists for procedural skills may be incomplete even regarding key components [39]. Future investigations should thus address conditions of changes in supervision levels related to distinct respective competencies as in the current literature this currently remains elusive. Additionally, we defined pre- and post-training external supervision levels by internal consultants’ discussion and consensus; hence no inter-rater reliability was assessed. Furthermore, we focused on short-term benefits of this skill training and data on long-term effects of this training are therefore not available. Importantly, retention of emergency skills is time-depended with training and repetition (e.g. every 6 months) likely required to sustain performance of emergency skills [40]. Further, focus of this investigation was on chest tube insertion, pericardiocentesis, and cricothyroidotomy as they may be regarded highly relevant and complex technical skills. Due to application in acute emergency situations, cost-benefit was not considered for pericardiocentesis and cricothyroidotomy. Moreover, chest tube insertion training appeared time saving when compared to traditional ICU-training approaches. However, as no standardized environment applied, this should be pursued in subsequent studies also.

## Conclusions

In ICU fellows, emergency skill training separated from daily clinical ICU-setting appeared useful to enhance learning experiences, skill performance, and levels of trust. Despite observed differences in self-reported and external assessments, external assessment of ICU-fellows observed a positive progression of performing emergency skills and led to reduced supervision levels.

## Data Availability

All data generated or analyzed during this study are included in this publication article.

## Abbreviations

ICU: Intensive Care Unit
SL: Supervision level
EPA: Entrustable professional activity
ECMO: Extracorporeal membrane oxygenation
CI: Confidence interval
IQR: Interquartile range
